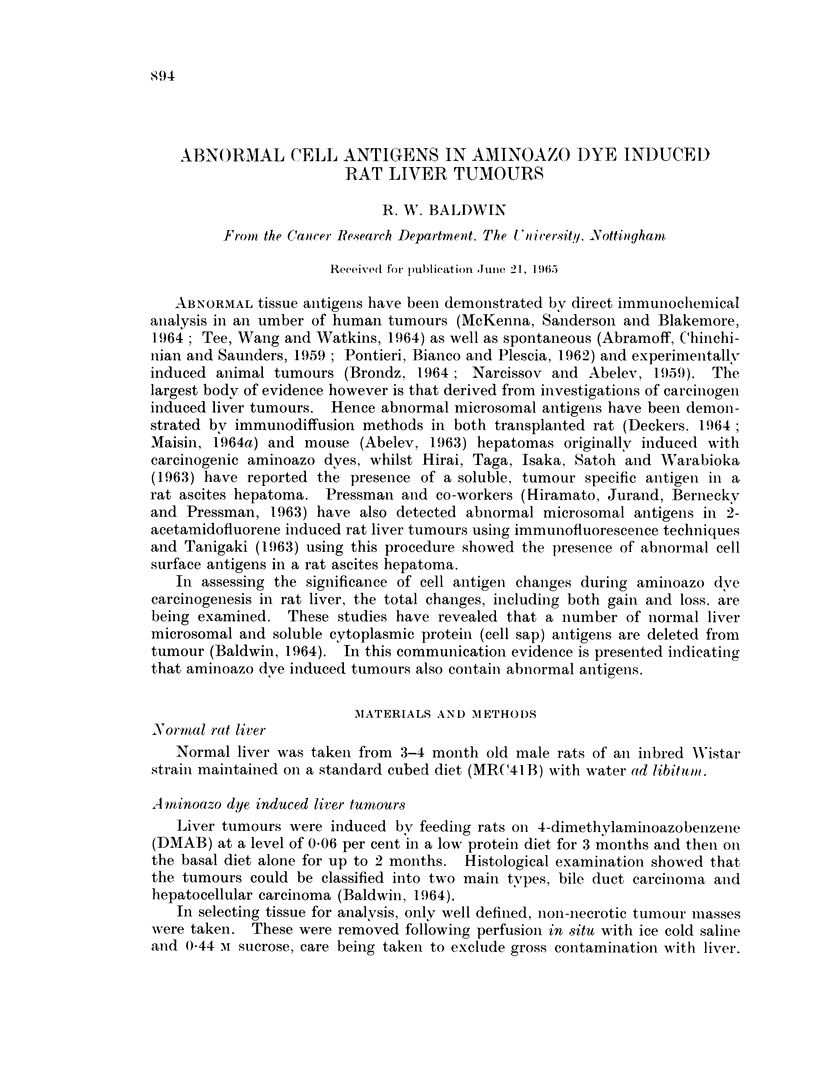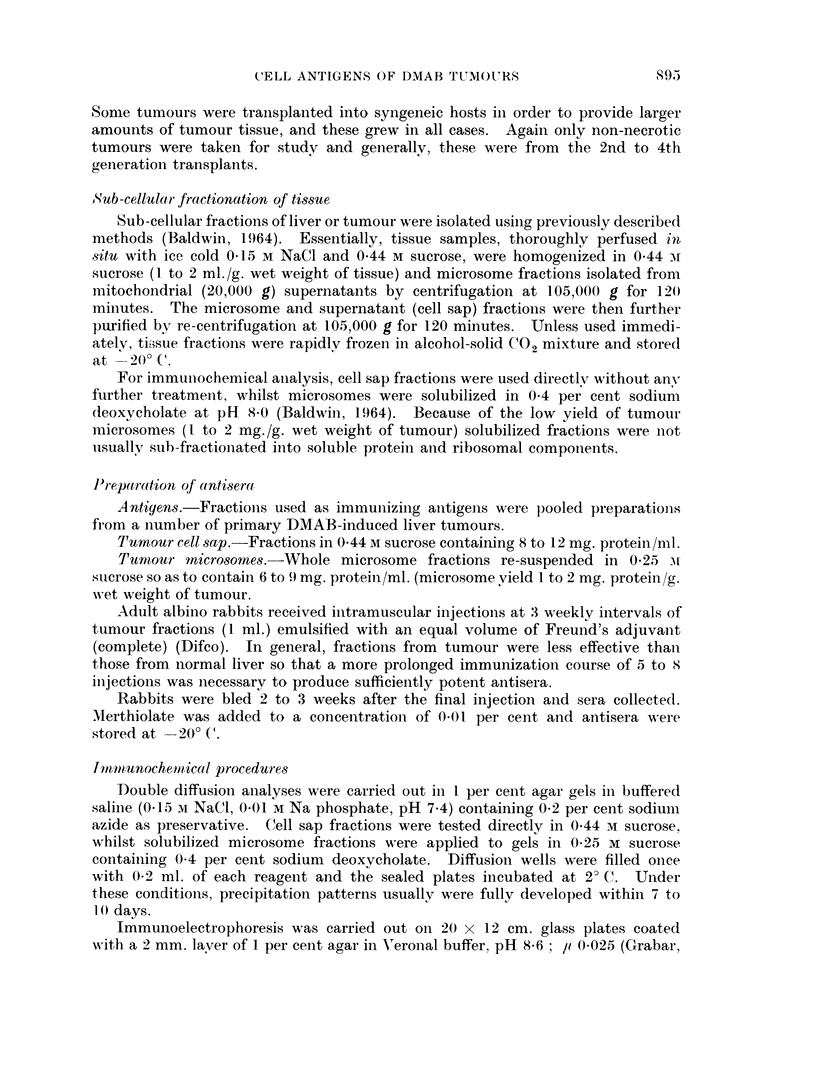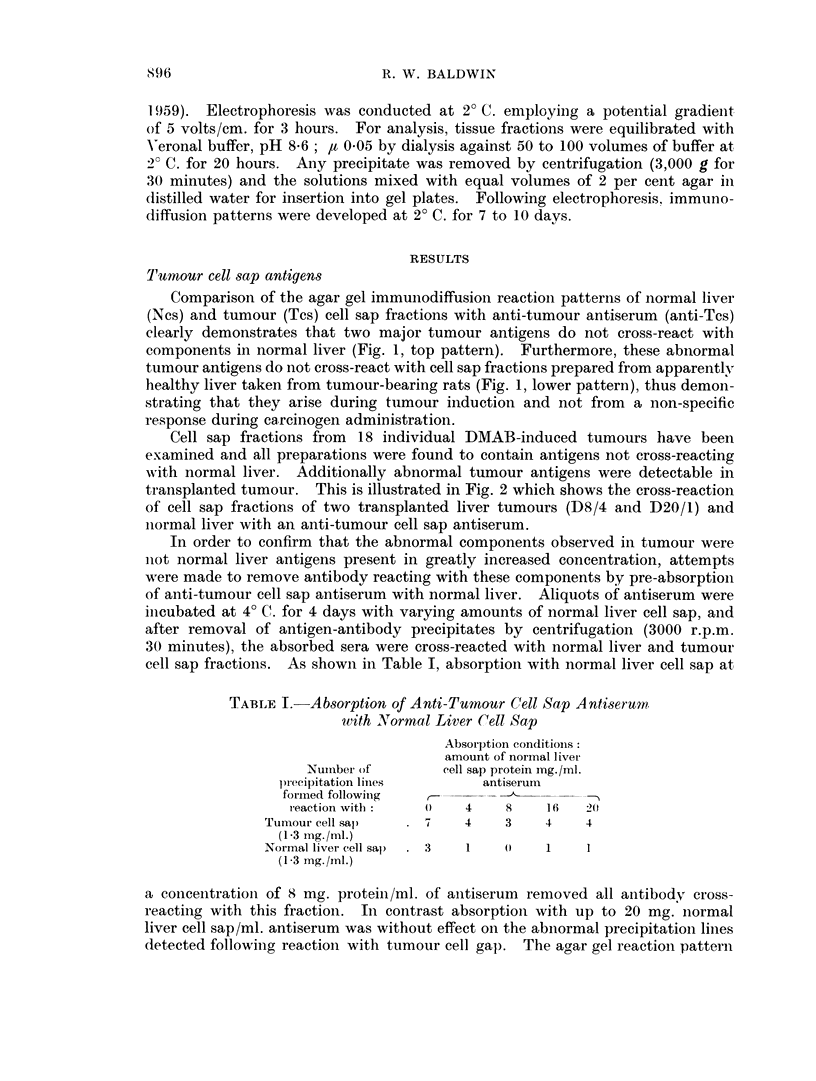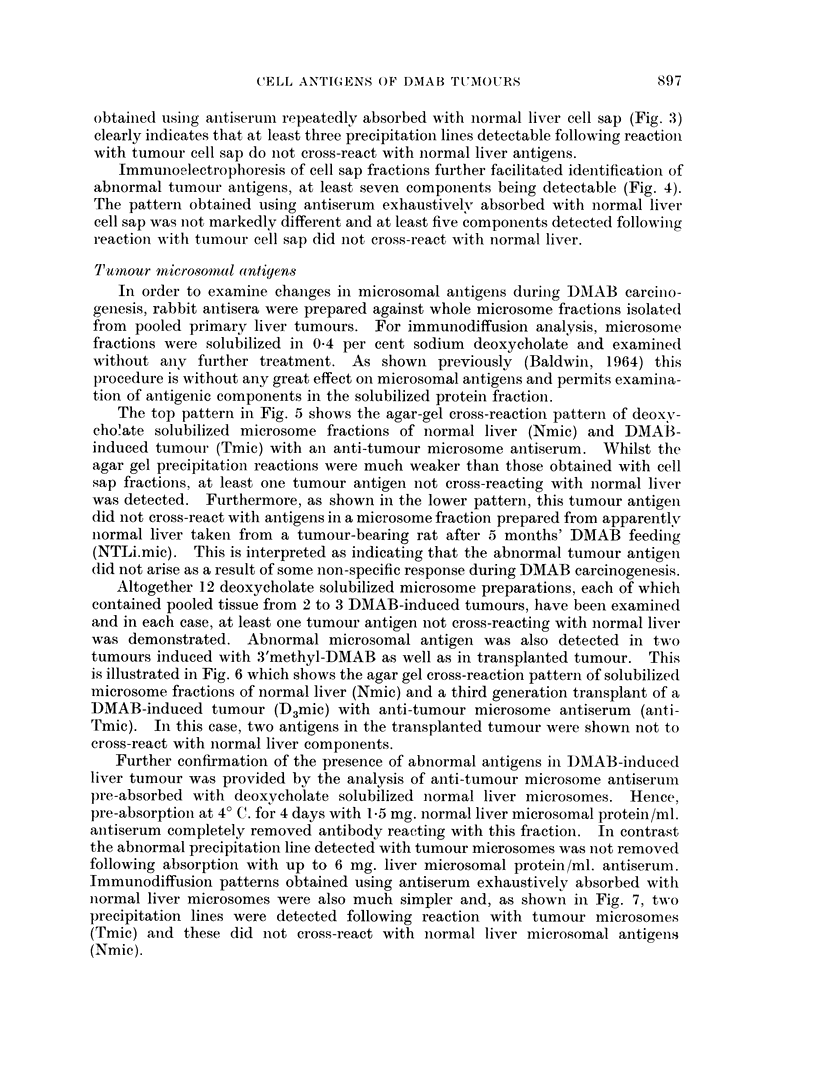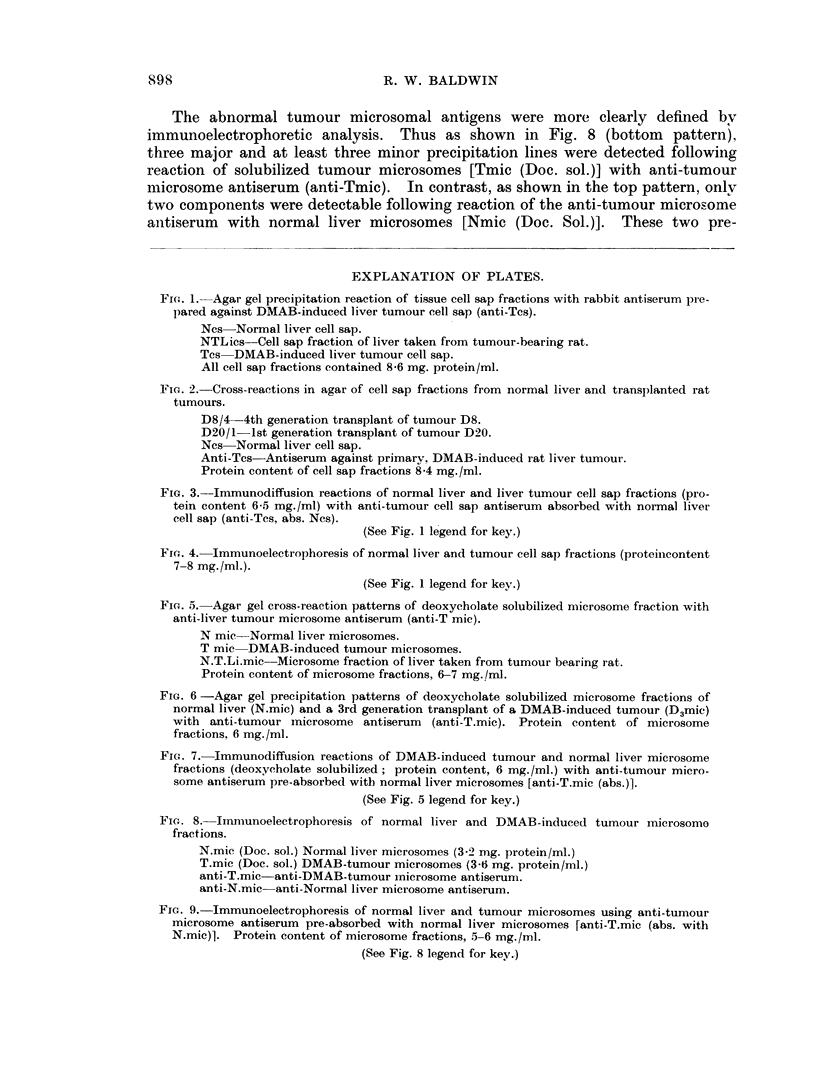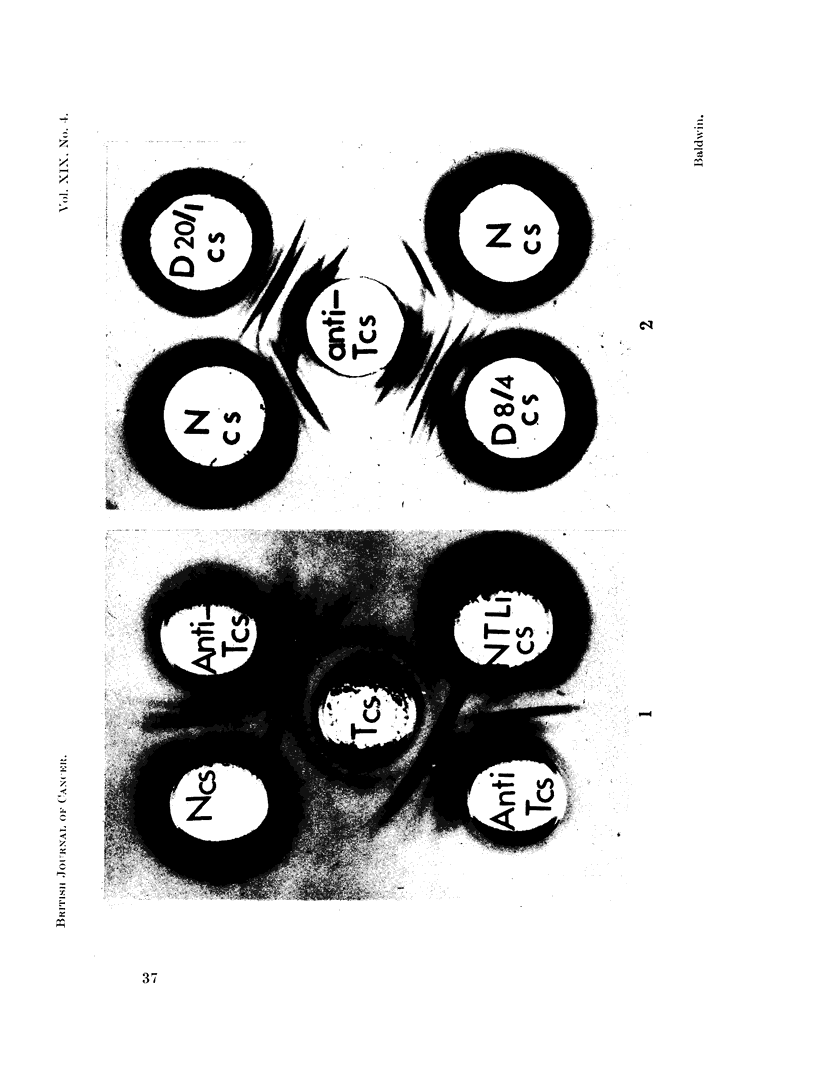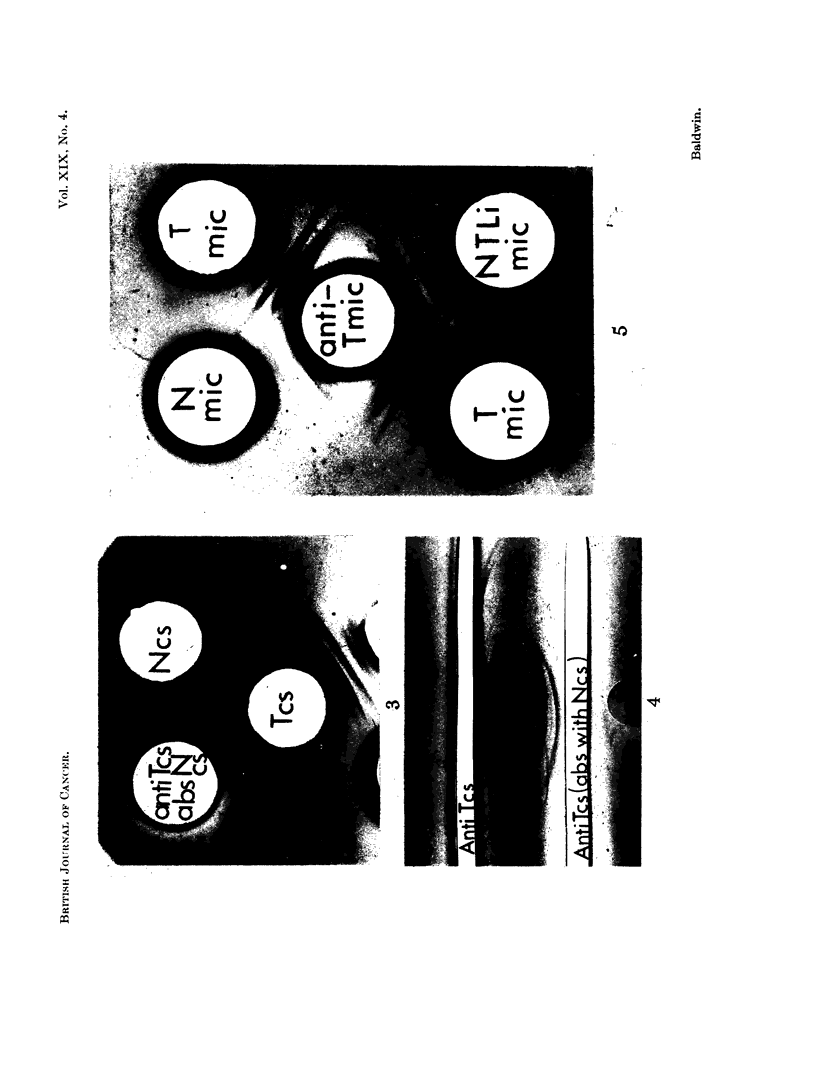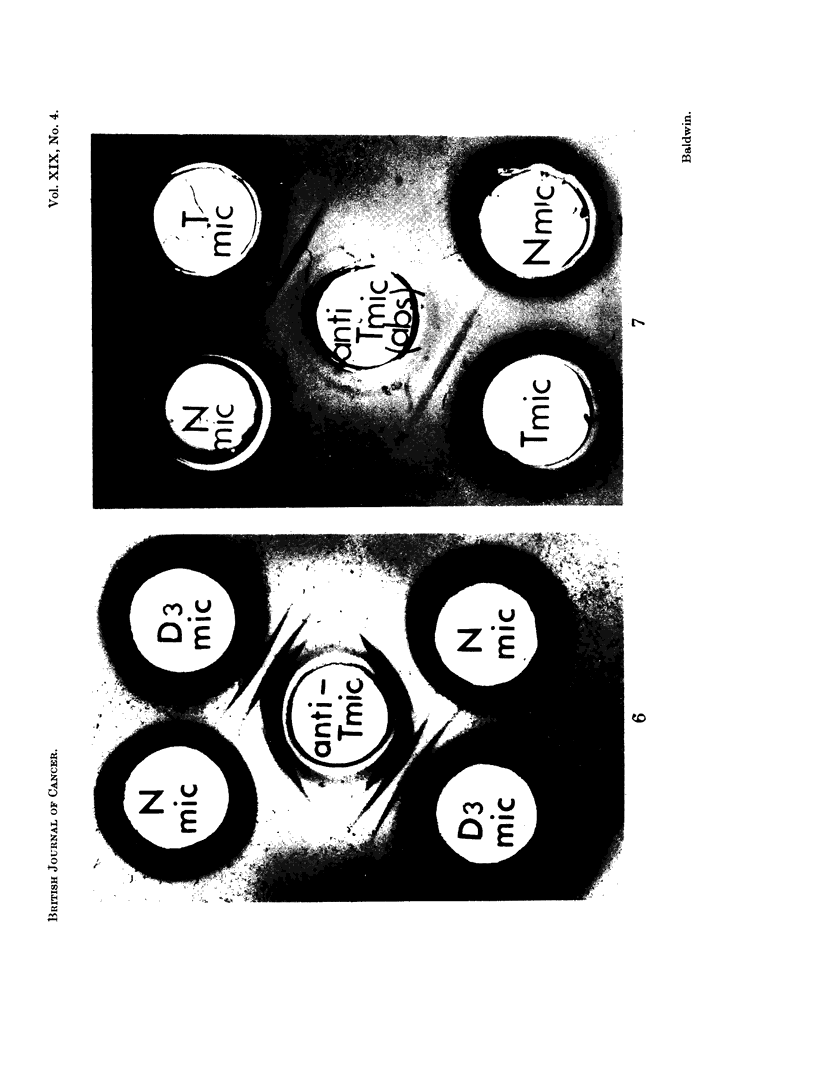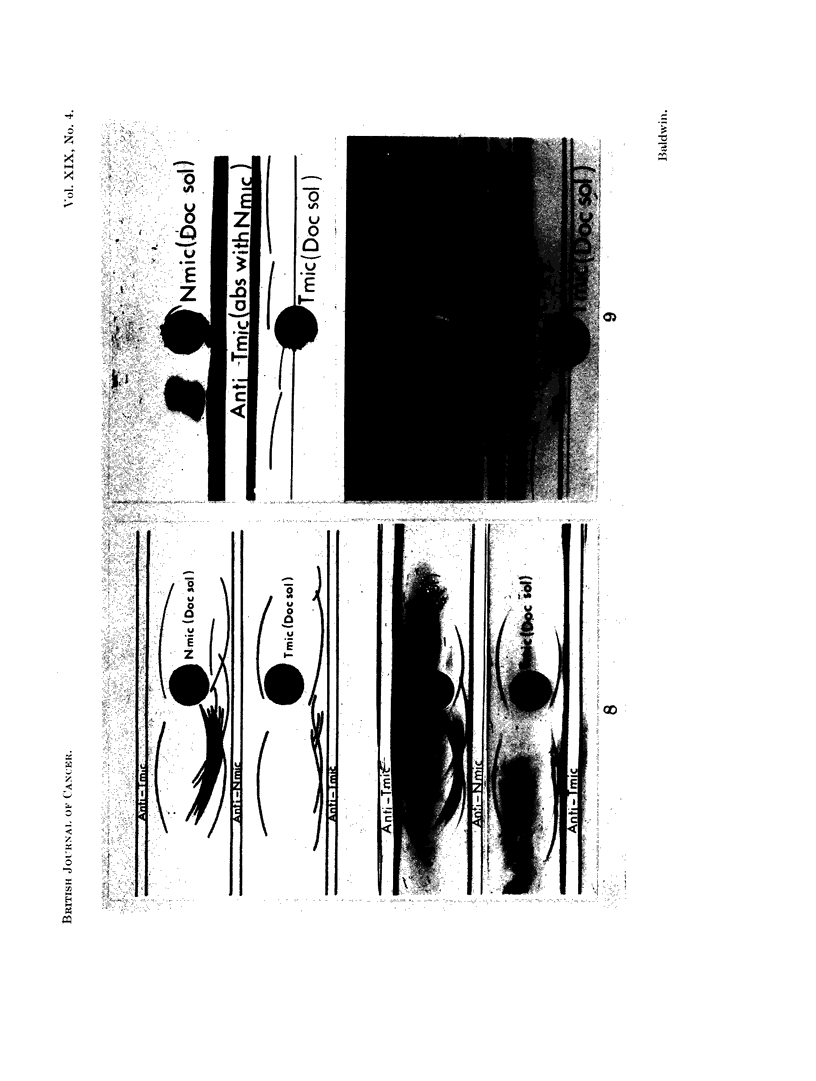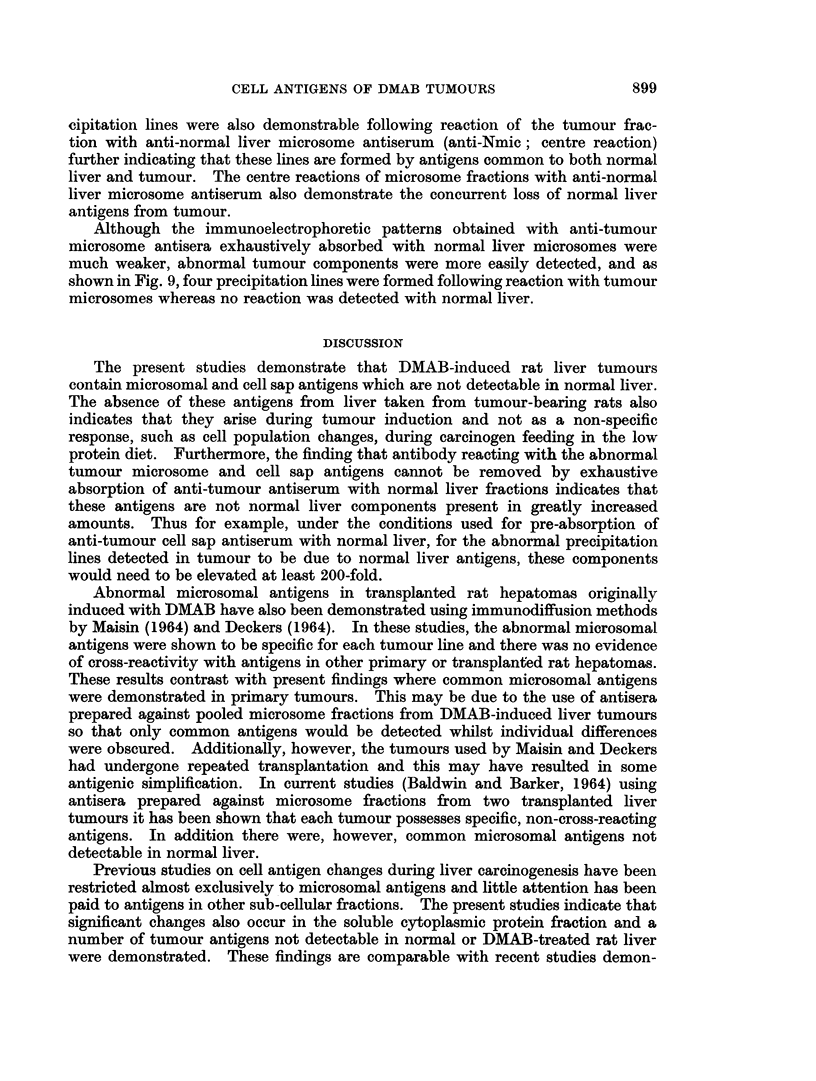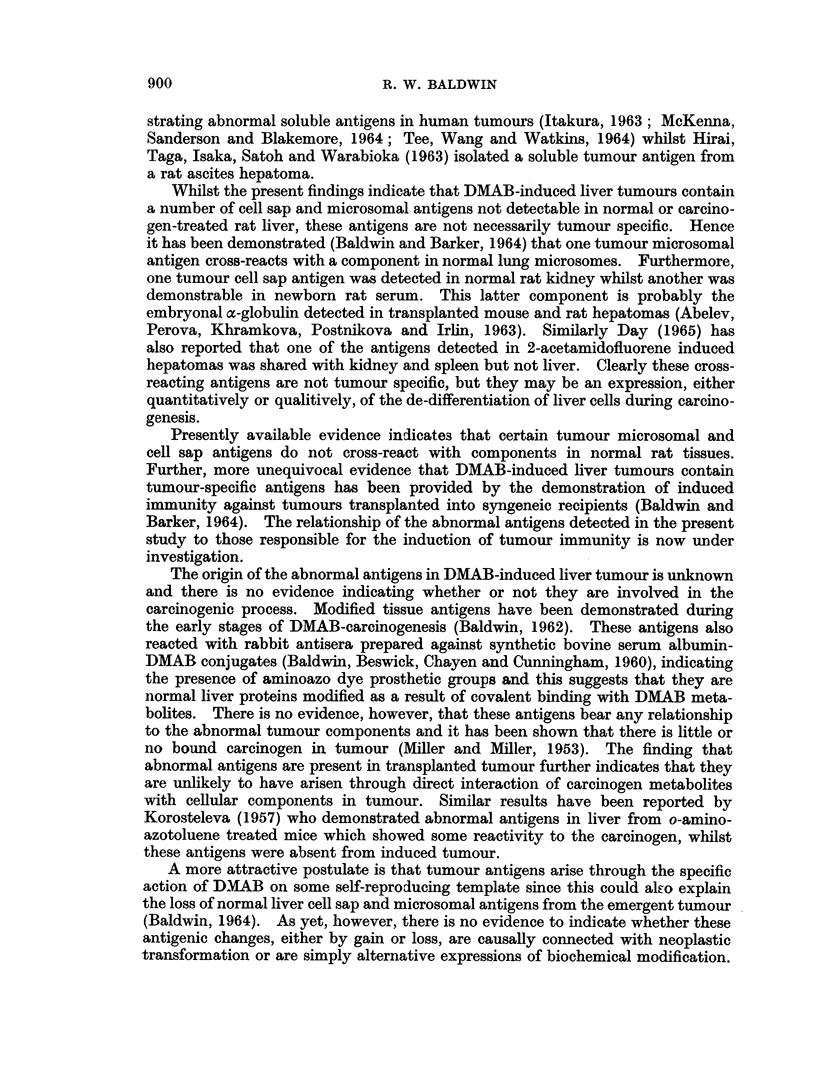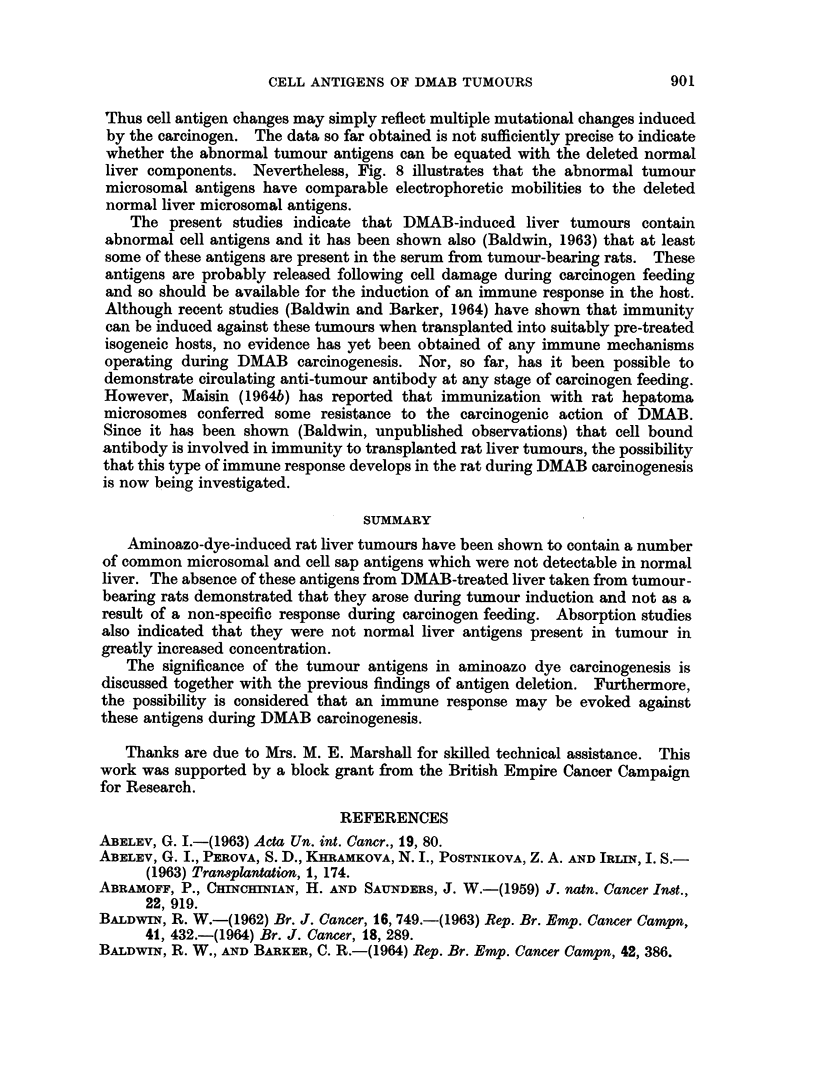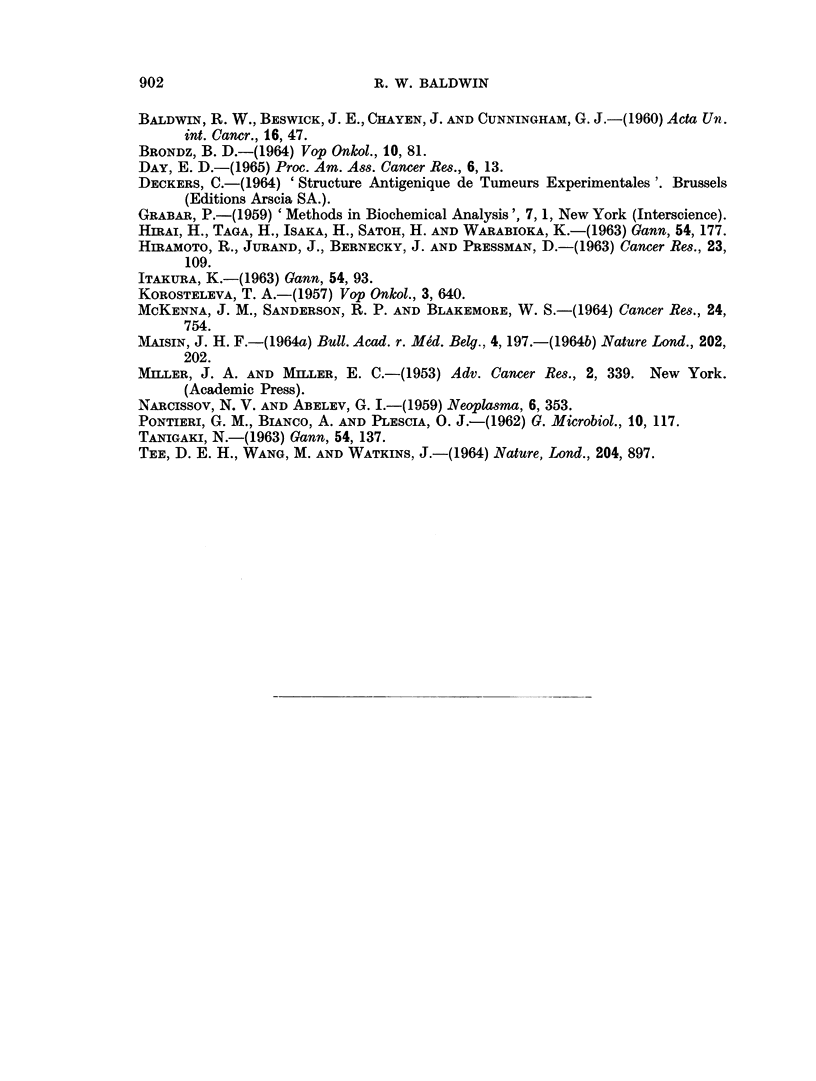# Abnormal cell antigens in aminoazo dye induced rat liver tumours.

**DOI:** 10.1038/bjc.1965.104

**Published:** 1965-12

**Authors:** R. W. Baldwin

## Abstract

**Images:**


### ABNORMAL CELL ANTIGENS IN AMINOAZO DYE INDUCED RAT LIVER TUMOURS

R. W. BALDWIN

*From the Cancer Research Department, The University, Nottingham*

Received for publication June 21, 1965

ABNORMAL tissue antigens have been demonstrated by direct immunochemical analysis in an umber of human tumours (McKenna, Sanderson and Blakemore, 1964 ; Tee, Wang and Watkins, 1964) as well as spontaneous (Abramoff, Chinchinian and Saunders, 1959 ; Pontieri, Bianco and Plescia, 1962) and experimentally induced animal tumours (Brondz, 1964 ; Narcissov and Abelev, 1959). The largest body of evidence however is that derived from investigations of carcinogen induced liver tumours. Hence abnormal microsomal antigens have been demonstrated by immunodiffusion methods in both transplanted rat (Deckers, 1964 ; Maisin, 1964*a*) and mouse (Abelev, 1963) hepatomas originally induced with carcinogenic aminoazo dyes, whilst Hirai, Taga, Isaka, Satoh and Warabioka (1963) have reported the presence of a soluble, tumour specific antigen in a rat ascites hepatoma. Pressman and co-workers (Hiramato, Jurand, Bernecky and Pressman, 1963) have also detected abnormal microsomal antigens in 2-acetamidofluorene induced rat liver tumours using immunofluorescence techniques and Tanigaki (1963) using this procedure showed the presence of abnormal cell surface antigens in a rat ascites hepatoma.

In assessing the significance of cell antigen changes during aminoazo dye carcinogenesis in rat liver, the total changes, including both gain and loss, are being examined. These studies have revealed that a number of normal liver microsomal and soluble cytoplasmic protein (cell sap) antigens are deleted from tumour (Baldwin, 1964). In this communication evidence is presented indicating that aminoazo dye induced tumours also contain abnormal antigens.

### MATERIALS AND METHODS

### *Normal rat liver*

Normal liver was taken from 3–4 month old male rats of an inbred Wistar strain maintained on a standard cubed diet (MRC41B) with water *ad libitum*.

### *Aminoazo dye induced liver tumours*

Liver tumours were induced by feeding rats on 4-dimethylaminoazobenzene (DMAB) at a level of 0·06 per cent in a low protein diet for 3 months and then on the basal diet alone for up to 2 months. Histological examination showed that the tumours could be classified into two main types, bile duct carcinoma and hepatocellular carcinoma (Baldwin, 1964).

In selecting tissue for analysis, only well defined, non-necrotic tumour masses were taken. These were removed following perfusion *in situ* with ice cold saline and 0·44 M sucrose, care being taken to exclude gross contamination with liver.

Some tumours were transplanted into syngeneic hosts in order to provide larger amounts of tumour tissue, and these grew in all cases. Again only non-necrotic tumours were taken for study and generally, these were from the 2nd to 4th generation transplants.

*Sub-cellular fractionation of tissue*

Sub-cellular fractions of liver or tumour were isolated using previously described methods (Baldwin, 1964). Essentially, tissue samples, thoroughly perfused *in situ* with ice cold 0·15 M NaCl and 0·44 M sucrose, were homogenized in 0·44 M sucrose (1 to 2 ml./g. wet weight of tissue) and microsome fractions isolated from mitochondrial (20,000 **g**) supernatants by centrifugation at 105,000 **g** for 120 minutes. The microsome and supernatant (cell sap) fractions were then further purified by re-centrifugation at 105,000 **g** for 120 minutes. Unless used immediately, tissue fractions were rapidly frozen in alcohol-solid $CO_2$ mixture and stored at −20° C.

For immunochemical analysis, cell sap fractions were used directly without any further treatment, whilst microsomes were solubilized in 0·4 per cent sodium deoxycholate at pH 8·0 (Baldwin, 1964). Because of the low yield of tumour microsomes (1 to 2 mg./g. wet weight of tumour) solubilized fractions were not usually sub-fractionated into soluble protein and ribosomal components.

*Preparation of antisera*

*Antigens.*—Fractions used as immunizing antigens were pooled preparations from a number of primary DMAB-induced liver tumours.

*Tumour cell sap.*—Fractions in 0·44 M sucrose containing 8 to 12 mg. protein/ml.

*Tumour microsomes.*—Whole microsome fractions re-suspended in 0·25 M sucrose so as to contain 6 to 9 mg. protein/ml. (microsome yield 1 to 2 mg. protein/g. wet weight of tumour.

Adult albino rabbits received intramuscular injections at 3 weekly intervals of tumour fractions (1 ml.) emulsified with an equal volume of Freund's adjuvant (complete) (Difco). In general, fractions from tumour were less effective than those from normal liver so that a more prolonged immunization course of 5 to 8 injections was necessary to produce sufficiently potent antisera.

Rabbits were bled 2 to 3 weeks after the final injection and sera collected. Merthiolate was added to a concentration of 0·01 per cent and antisera were stored at −20° C.

*Immunochemical procedures*

Double diffusion analyses were carried out in 1 per cent agar gels in buffered saline (0·15 M NaCl, 0·01 M Na phosphate, pH 7·4) containing 0·2 per cent sodium azide as preservative. Cell sap fractions were tested directly in 0·44 M sucrose, whilst solubilized microsome fractions were applied to gels in 0·25 M sucrose containing 0·4 per cent sodium deoxycholate. Diffusion wells were filled once with 0·2 ml. of each reagent and the sealed plates incubated at 2° C. Under these conditions, precipitation patterns usually were fully developed within 7 to 10 days.

Immunoelectrophoresis was carried out on 20 × 12 cm. glass plates coated with a 2 mm. layer of 1 per cent agar in Veronal buffer, pH 8·6 ; $\mu$ 0·025 (Grabar,

1959). Electrophoresis was conducted at 2° C. employing a potential gradient of 5 volts/cm. for 3 hours. For analysis, tissue fractions were equilibrated with Veronal buffer, pH 8·6 ; $\mu$ 0·05 by dialysis against 50 to 100 volumes of buffer at 2° C. for 20 hours. Any precipitate was removed by centrifugation (3,000 *g* for 30 minutes) and the solutions mixed with equal volumes of 2 per cent agar in distilled water for insertion into gel plates. Following electrophoresis, immunodiffusion patterns were developed at 2° C. for 7 to 10 days.

### RESULTS

*Tumour cell sap antigens*

Comparison of the agar gel immunodiffusion reaction patterns of normal liver (Ncs) and tumour (Tcs) cell sap fractions with anti-tumour antiserum (anti-Tcs) clearly demonstrates that two major tumour antigens do not cross-react with components in normal liver (Fig. 1, top pattern). Furthermore, these abnormal tumour antigens do not cross-react with cell sap fractions prepared from apparently healthy liver taken from tumour-bearing rats (Fig. 1, lower pattern), thus demonstrating that they arise during tumour induction and not from a non-specific response during carcinogen administration.

Cell sap fractions from 18 individual DMAB-induced tumours have been examined and all preparations were found to contain antigens not cross-reacting with normal liver. Additionally abnormal tumour antigens were detectable in transplanted tumour. This is illustrated in Fig. 2 which shows the cross-reaction of cell sap fractions of two transplanted liver tumours (D8/4 and D20/1) and normal liver with an anti-tumour cell sap antiserum.

In order to confirm that the abnormal components observed in tumour were not normal liver antigens present in greatly increased concentration, attempts were made to remove antibody reacting with these components by pre-absorption of anti-tumour cell sap antiserum with normal liver. Aliquots of antiserum were incubated at 4° C. for 4 days with varying amounts of normal liver cell sap, and after removal of antigen-antibody precipitates by centrifugation (3000 r.p.m. 30 minutes), the absorbed sera were cross-reacted with normal liver and tumour cell sap fractions. As shown in Table I, absorption with normal liver cell sap at

TABLE I.—*Absorption of Anti-Tumour Cell Sap Antiserum with Normal Liver Cell Sap*

| Number of precipitation lines formed following reaction with : | Absorption conditions : amount of normal liver cell sap protein mg./ml. antiserum | | | | |
|---|---|---|---|---|---|
| | 0 | 4 | 8 | 16 | 20 |
| Tumour cell sap (1·3 mg./ml.) | 7 | 4 | 3 | 4 | 4 |
| Normal liver cell sap (1·3 mg./ml.) | 3 | 1 | 0 | 1 | 1 |

a concentration of 8 mg. protein/ml. of antiserum removed all antibody cross-reacting with this fraction. In contrast absorption with up to 20 mg. normal liver cell sap/ml. antiserum was without effect on the abnormal precipitation lines detected following reaction with tumour cell gap. The agar gel reaction pattern obtained using antiserum repeatedly absorbed with normal liver cell sap (Fig. 3) clearly indicates that at least three precipitation lines detectable following reaction with tumour cell sap do not cross-react with normal liver antigens.

Immunoelectrophoresis of cell sap fractions further facilitated identification of abnormal tumour antigens, at least seven components being detectable (Fig. 4). The pattern obtained using antiserum exhaustively absorbed with normal liver cell sap was not markedly different and at least five components detected following reaction with tumour cell sap did not cross-react with normal liver.

*Tumour microsomal antigens*

In order to examine changes in microsomal antigens during DMAB carcinogenesis, rabbit antisera were prepared against whole microsome fractions isolated from pooled primary liver tumours. For immunodiffusion analysis, microsome fractions were solubilized in 0·4 per cent sodium deoxycholate and examined without any further treatment. As shown previously (Baldwin, 1964) this procedure is without any great effect on microsomal antigens and permits examination of antigenic components in the solubilized protein fraction.

The top pattern in Fig. 5 shows the agar-gel cross-reaction pattern of deoxycholate solubilized microsome fractions of normal liver (Nmic) and DMAB-induced tumour (Tmic) with an anti-tumour microsome antiserum. Whilst the agar gel precipitation reactions were much weaker than those obtained with cell sap fractions, at least one tumour antigen not cross-reacting with normal liver was detected. Furthermore, as shown in the lower pattern, this tumour antigen did not cross-react with antigens in a microsome fraction prepared from apparently normal liver taken from a tumour-bearing rat after 5 months' DMAB feeding (NTLi.mic). This is interpreted as indicating that the abnormal tumour antigen did not arise as a result of some non-specific response during DMAB carcinogenesis.

Altogether 12 deoxycholate solubilized microsome preparations, each of which contained pooled tissue from 2 to 3 DMAB-induced tumours, have been examined and in each case, at least one tumour antigen not cross-reacting with normal liver was demonstrated. Abnormal microsomal antigen was also detected in two tumours induced with 3'methyl-DMAB as well as in transplanted tumour. This is illustrated in Fig. 6 which shows the agar gel cross-reaction pattern of solubilized microsome fractions of normal liver (Nmic) and a third generation transplant of a DMAB-induced tumour ($D_3$mic) with anti-tumour microsome antiserum (anti-Tmic). In this case, two antigens in the transplanted tumour were shown not to cross-react with normal liver components.

Further confirmation of the presence of abnormal antigens in DMAB-induced liver tumour was provided by the analysis of anti-tumour microsome antiserum pre-absorbed with deoxycholate solubilized normal liver microsomes. Hence, pre-absorption at 4° C. for 4 days with 1·5 mg. normal liver microsomal protein/ml. antiserum completely removed antibody reacting with this fraction. In contrast the abnormal precipitation line detected with tumour microsomes was not removed following absorption with up to 6 mg. liver microsomal protein/ml. antiserum. Immunodiffusion patterns obtained using antiserum exhaustively absorbed with normal liver microsomes were also much simpler and, as shown in Fig. 7, two precipitation lines were detected following reaction with tumour microsomes (Tmic) and these did not cross-react with normal liver microsomal antigens (Nmic).

The abnormal tumour microsomal antigens were more clearly defined by immunoelectrophoretic analysis. Thus as shown in Fig. 8 (bottom pattern), three major and at least three minor precipitation lines were detected following reaction of solubilized tumour microsomes [Tmic (Doc. sol.)] with anti-tumour microsome antiserum (anti-Tmic). In contrast, as shown in the top pattern, only two components were detectable following reaction of the anti-tumour microsome antiserum with normal liver microsomes [Nmic (Doc. Sol.)]. These two pre-

---

### EXPLANATION OF PLATES.

FIG. 1.—Agar gel precipitation reaction of tissue cell sap fractions with rabbit antiserum prepared against DMAB-induced liver tumour cell sap (anti-Tcs).

Ncs—Normal liver cell sap.
NTLics—Cell sap fraction of liver taken from tumour-bearing rat.
Tcs—DMAB-induced liver tumour cell sap.
All cell sap fractions contained 8·6 mg. protein/ml.

FIG. 2.—Cross-reactions in agar of cell sap fractions from normal liver and transplanted rat tumours.

D8/4—4th generation transplant of tumour D8.
D20/1—1st generation transplant of tumour D20.
Ncs—Normal liver cell sap.
Anti-Tcs—Antiserum against primary, DMAB-induced rat liver tumour.
Protein content of cell sap fractions 8·4 mg./ml.

FIG. 3.—Immunodiffusion reactions of normal liver and liver tumour cell sap fractions (protein content 6·5 mg./ml) with anti-tumour cell sap antiserum absorbed with normal liver cell sap (anti-Tcs, abs. Ncs).

(See Fig. 1 legend for key.)

FIG. 4.—Immunoelectrophoresis of normal liver and tumour cell sap fractions (proteincontent 7–8 mg./ml.).

(See Fig. 1 legend for key.)

FIG. 5.—Agar gel cross-reaction patterns of deoxycholate solubilized microsome fraction with anti-liver tumour microsome antiserum (anti-T mic).

N mic—Normal liver microsomes.
T mic—DMAB-induced tumour microsomes.
N.T.Li.mic—Microsome fraction of liver taken from tumour bearing rat.
Protein content of microsome fractions, 6–7 mg./ml.

FIG. 6 —Agar gel precipitation patterns of deoxycholate solubilized microsome fractions of normal liver (N.mic) and a 3rd generation transplant of a DMAB-induced tumour ($D_3$mic) with anti-tumour microsome antiserum (anti-T.mic). Protein content of microsome fractions, 6 mg./ml.

FIG. 7.—Immunodiffusion reactions of DMAB-induced tumour and normal liver microsome fractions (deoxycholate solubilized ; protein content, 6 mg./ml.) with anti-tumour microsome antiserum pre-absorbed with normal liver microsomes [anti-T.mic (abs.)].

(See Fig. 5 legend for key.)

FIG. 8.—Immunoelectrophoresis of normal liver and DMAB-induced tumour microsome fractions.

N.mic (Doc. sol.) Normal liver microsomes (3·2 mg. protein/ml.)
T.mic (Doc. sol.) DMAB-tumour microsomes (3·6 mg. protein/ml.)
anti-T.mic—anti-DMAB-tumour microsome antiserum.
anti-N.mic—anti-Normal liver microsome antiserum.

FIG. 9.—Immunoelectrophoresis of normal liver and tumour microsomes using anti-tumour microsome antiserum pre-absorbed with normal liver microsomes [anti-T.mic (abs. with N.mic)]. Protein content of microsome fractions, 5–6 mg./ml.

(See Fig. 8 legend for key.)

Baldwin.

cipitation lines were also demonstrable following reaction of the tumour fraction with anti-normal liver microsome antiserum (anti-Nmic ; centre reaction) further indicating that these lines are formed by antigens common to both normal liver and tumour. The centre reactions of microsome fractions with anti-normal liver microsome antiserum also demonstrate the concurrent loss of normal liver antigens from tumour.

Although the immunoelectrophoretic patterns obtained with anti-tumour microsome antisera exhaustively absorbed with normal liver microsomes were much weaker, abnormal tumour components were more easily detected, and as shown in Fig. 9, four precipitation lines were formed following reaction with tumour microsomes whereas no reaction was detected with normal liver.

### DISCUSSION

The present studies demonstrate that DMAB-induced rat liver tumours contain microsomal and cell sap antigens which are not detectable in normal liver. The absence of these antigens from liver taken from tumour-bearing rats also indicates that they arise during tumour induction and not as a non-specific response, such as cell population changes, during carcinogen feeding in the low protein diet. Furthermore, the finding that antibody reacting with the abnormal tumour microsome and cell sap antigens cannot be removed by exhaustive absorption of anti-tumour antiserum with normal liver fractions indicates that these antigens are not normal liver components present in greatly increased amounts. Thus for example, under the conditions used for pre-absorption of anti-tumour cell sap antiserum with normal liver, for the abnormal precipitation lines detected in tumour to be due to normal liver antigens, these components would need to be elevated at least 200-fold.

Abnormal microsomal antigens in transplanted rat hepatomas originally induced with DMAB have also been demonstrated using immunodiffusion methods by Maisin (1964) and Deckers (1964). In these studies, the abnormal microsomal antigens were shown to be specific for each tumour line and there was no evidence of cross-reactivity with antigens in other primary or transplanted rat hepatomas. These results contrast with present findings where common microsomal antigens were demonstrated in primary tumours. This may be due to the use of antisera prepared against pooled microsome fractions from DMAB-induced liver tumours so that only common antigens would be detected whilst individual differences were obscured. Additionally, however, the tumours used by Maisin and Deckers had undergone repeated transplantation and this may have resulted in some antigenic simplification. In current studies (Baldwin and Barker, 1964) using antisera prepared against microsome fractions from two transplanted liver tumours it has been shown that each tumour possesses specific, non-cross-reacting antigens. In addition there were, however, common microsomal antigens not detectable in normal liver.

Previous studies on cell antigen changes during liver carcinogenesis have been restricted almost exclusively to microsomal antigens and little attention has been paid to antigens in other sub-cellular fractions. The present studies indicate that significant changes also occur in the soluble cytoplasmic protein fraction and a number of tumour antigens not detectable in normal or DMAB-treated rat liver were demonstrated. These findings are comparable with recent studies demonstrating abnormal soluble antigens in human tumours (Itakura, 1963 ; McKenna, Sanderson and Blakemore, 1964 ; Tee, Wang and Watkins, 1964) whilst Hirai, Taga, Isaka, Satoh and Warabioka (1963) isolated a soluble tumour antigen from a rat ascites hepatoma.

Whilst the present findings indicate that DMAB-induced liver tumours contain a number of cell sap and microsomal antigens not detectable in normal or carcinogen-treated rat liver, these antigens are not necessarily tumour specific. Hence it has been demonstrated (Baldwin and Barker, 1964) that one tumour microsomal antigen cross-reacts with a component in normal lung microsomes. Furthermore, one tumour cell sap antigen was detected in normal rat kidney whilst another was demonstrable in newborn rat serum. This latter component is probably the embryonal $\alpha$-globulin detected in transplanted mouse and rat hepatomas (Abelev, Perova, Khramkova, Postnikova and Irlin, 1963). Similarly Day (1965) has also reported that one of the antigens detected in 2-acetamidofluorene induced hepatomas was shared with kidney and spleen but not liver. Clearly these cross-reacting antigens are not tumour specific, but they may be an expression, either quantitatively or qualitively, of the de-differentiation of liver cells during carcinogenesis.

Presently available evidence indicates that certain tumour microsomal and cell sap antigens do not cross-react with components in normal rat tissues. Further, more unequivocal evidence that DMAB-induced liver tumours contain tumour-specific antigens has been provided by the demonstration of induced immunity against tumours transplanted into syngeneic recipients (Baldwin and Barker, 1964). The relationship of the abnormal antigens detected in the present study to those responsible for the induction of tumour immunity is now under investigation.

The origin of the abnormal antigens in DMAB-induced liver tumour is unknown and there is no evidence indicating whether or not they are involved in the carcinogenic process. Modified tissue antigens have been demonstrated during the early stages of DMAB-carcinogenesis (Baldwin, 1962). These antigens also reacted with rabbit antisera prepared against synthetic bovine serum albumin-DMAB conjugates (Baldwin, Beswick, Chayen and Cunningham, 1960), indicating the presence of aminoazo dye prosthetic groups and this suggests that they are normal liver proteins modified as a result of covalent binding with DMAB metabolites. There is no evidence, however, that these antigens bear any relationship to the abnormal tumour components and it has been shown that there is little or no bound carcinogen in tumour (Miller and Miller, 1953). The finding that abnormal antigens are present in transplanted tumour further indicates that they are unlikely to have arisen through direct interaction of carcinogen metabolites with cellular components in tumour. Similar results have been reported by Korosteleva (1957) who demonstrated abnormal antigens in liver from *o*-aminoazotoluene treated mice which showed some reactivity to the carcinogen, whilst these antigens were absent from induced tumour.

A more attractive postulate is that tumour antigens arise through the specific action of DMAB on some self-reproducing template since this could also explain the loss of normal liver cell sap and microsomal antigens from the emergent tumour (Baldwin, 1964). As yet, however, there is no evidence to indicate whether these antigenic changes, either by gain or loss, are causally connected with neoplastic transformation or are simply alternative expressions of biochemical modification.

Thus cell antigen changes may simply reflect multiple mutational changes induced by the carcinogen. The data so far obtained is not sufficiently precise to indicate whether the abnormal tumour antigens can be equated with the deleted normal liver components. Nevertheless, Fig. 8 illustrates that the abnormal tumour microsomal antigens have comparable electrophoretic mobilities to the deleted normal liver microsomal antigens.

The present studies indicate that DMAB-induced liver tumours contain abnormal cell antigens and it has been shown also (Baldwin, 1963) that at least some of these antigens are present in the serum from tumour-bearing rats. These antigens are probably released following cell damage during carcinogen feeding and so should be available for the induction of an immune response in the host. Although recent studies (Baldwin and Barker, 1964) have shown that immunity can be induced against these tumours when transplanted into suitably pre-treated isogeneic hosts, no evidence has yet been obtained of any immune mechanisms operating during DMAB carcinogenesis. Nor, so far, has it been possible to demonstrate circulating anti-tumour antibody at any stage of carcinogen feeding. However, Maisin (1964*b*) has reported that immunization with rat hepatoma microsomes conferred some resistance to the carcinogenic action of DMAB. Since it has been shown (Baldwin, unpublished observations) that cell bound antibody is involved in immunity to transplanted rat liver tumours, the possibility that this type of immune response develops in the rat during DMAB carcinogenesis is now being investigated.

### SUMMARY

Aminoazo-dye-induced rat liver tumours have been shown to contain a number of common microsomal and cell sap antigens which were not detectable in normal liver. The absence of these antigens from DMAB-treated liver taken from tumour-bearing rats demonstrated that they arose during tumour induction and not as a result of a non-specific response during carcinogen feeding. Absorption studies also indicated that they were not normal liver antigens present in tumour in greatly increased concentration.

The significance of the tumour antigens in aminoazo dye carcinogenesis is discussed together with the previous findings of antigen deletion. Furthermore, the possibility is considered that an immune response may be evoked against these antigens during DMAB carcinogenesis.

Thanks are due to Mrs. M. E. Marshall for skilled technical assistance. This work was supported by a block grant from the British Empire Cancer Campaign for Research.

### REFERENCES

ABELEV, G. I.—(1963) *Acta Un. int. Cancr.*, **19**, 80.

ABELEV, G. I., PEROVA, S. D., KHRAMKOVA, N. I., POSTNIKOVA, Z. A. AND IRLIN, I. S.—(1963) *Transplantation*, **1**, 174.

ABRAMOFF, P., CHINCHINIAN, H. AND SAUNDERS, J. W.—(1959) *J. natn. Cancer Inst.*, **22**, 919.

BALDWIN, R. W.—(1962) *Br. J. Cancer*, **16**, 749.—(1963) *Rep. Br. Emp. Cancer Campn*, **41**, 432.—(1964) *Br. J. Cancer*, **18**, 289.

BALDWIN, R. W., AND BARKER, C. R.—(1964) *Rep. Br. Emp. Cancer Campn*, **42**, 386.

BALDWIN, R. W., BESWICK, J. E., CHAYEN, J. AND CUNNINGHAM, G. J.—(1960) *Acta Un. int. Cancr.*, **16**, 47.
BRONDZ, B. D.—(1964) *Vop Onkol.*, **10**, 81.
DAY, E. D.—(1965) *Proc. Am. Ass. Cancer Res.*, **6**, 13.
DECKERS, C.—(1964) 'Structure Antigenique de Tumeurs Experimentales'. Brussels (Editions Arscia SA.).
GRABAR, P.—(1959) 'Methods in Biochemical Analysis', **7**, 1, New York (Interscience).
HIRAI, H., TAGA, H., ISAKA, H., SATOH, H. AND WARABIOKA, K.—(1963) *Gann*, **54**, 177.
HIRAMOTO, R., JURAND, J., BERNECKY, J. AND PRESSMAN, D.—(1963) *Cancer Res.*, **23**, 109.
ITAKURA, K.—(1963) *Gann*, **54**, 93.
KOROSTELEVA, T. A.—(1957) *Vop Onkol.*, **3**, 640.
MCKENNA, J. M., SANDERSON, R. P. AND BLAKEMORE, W. S.—(1964) *Cancer Res.*, **24**, 754.
MAISIN, J. H. F.—(1964*a*) *Bull. Acad. r. Méd. Belg.*, **4**, 197.—(1964*b*) *Nature Lond.*, **202**, 202.
MILLER, J. A. AND MILLER, E. C.—(1953) *Adv. Cancer Res.*, **2**, 339. New York. (Academic Press).
NARCISSOV, N. V. AND ABELEV, G. I.—(1959) *Neoplasma*, **6**, 353.
PONTIERI, G. M., BIANCO, A. AND PLESCIA, O. J.—(1962) *G. Microbiol.*, **10**, 117.
TANIGAKI, N.—(1963) *Gann*, **54**, 137.
TEE, D. E. H., WANG, M. AND WATKINS, J.—(1964) *Nature, Lond.*, **204**, 897.